# SNAPshots of the MCHR1: a Comparison Between the PET-Tracers [^18^F]FE@SNAP and [^11^C]SNAP-7941

**DOI:** 10.1007/s11307-018-1212-0

**Published:** 2018-06-13

**Authors:** Cécile Philippe, Markus Zeilinger, Monika Dumanic, Florian Pichler, Lukas Fetty, Chrysoula Vraka, Theresa Balber, Wolfgang Wadsak, Katharina Pallitsch, Helmut Spreitzer, Rupert Lanzenberger, Marcus Hacker, Markus Mitterhauser

**Affiliations:** 1 Department of Biomedical Imaging and Image-guided Therapy, Division of Nuclear Medicine, Medical University of Vienna, Waehringer Guertel 18-20, 1090 Vienna, Austria; 2 Department of Pharmaceutical Technology and Biopharmaceutics, University of Vienna, Vienna, Austria; 3 Faculty of Engineering, University of Applied Sciences Wiener Neustadt, Neustadt, Austria; 4 Department of Radiotherapy, Division of Medical Physics, Medical University of Vienna, Vienna, Austria; 5 Department of Inorganic Chemistry, University of Vienna, Vienna, Austria; 6 CBmed, Graz, Austria; 7 Department of Organic Chemistry, University of Vienna, Vienna, Austria; 8 Department of Pharmaceutical Chemistry, University of Vienna, Vienna, Austria; 9 Department of Psychiatry and Psychotherapy, Medical University of Vienna, Vienna, Austria; 10 Ludwig Boltzmann Institute Applied Diagnostics, Vienna, Austria

**Keywords:** [^11^C]SNAP-7941, [^18^F]FE@SNAP, MCHR1, PET, Small animal imaging, *In vitro*, *In vivo*, *Ex vivo*

## Abstract

**Purpose:**

The melanin-concentrating hormone receptor 1 (MCHR1) has become an important pharmacological target, since it may be involved in various diseases, such as diabetes, insulin resistance, and obesity. Hence, a suitable positron emission tomography radiotracer for the *in vivo* assessment of the MCHR1 pharmacology is imperative. The current paper contrasts the extensive *in vitro*, *in vivo*, and *ex vivo* assessments of the radiotracers [^18^F]FE@SNAP and [^11^C]SNAP-7941 and provides comprehensive information about their biological and physicochemical properties. Furthermore, it examines their suitability for first-in-man imaging studies.

**Procedures:**

Kinetic real-time cell-binding studies with [^18^F]FE@SNAP and [^11^C]SNAP-7941 were conducted on adherent Chines hamster ovary (CHO-K1) cells stably expressing the human MCHR1 and MCHR2. Small animal imaging studies on mice and rats were performed under displacement and baseline conditions, as well as after pretreatment with the P-glycoprotein/breast cancer resistant protein inhibitor tariquidar. After the imaging studies, detailed analyses of the *ex vivo* biodistribution were performed. *Ex vivo* metabolism was determined in rat blood and brain and analyzed at various time points using a quantitative radio-HPLC assay.

**Results:**

[^11^C]SNAP-7941 demonstrates high uptake on CHO-K1-hMCHR1 cells, whereas no uptake was detected for the CHO-K1-hMCHR2 cells. In contrast, [^18^F]FE@SNAP evinced binding to CHO-K1-hMCHR1 and CHO-K1-hMCHR2 cells. Imaging studies with [^18^F]FE@SNAP and [^11^C]SNAP-7941 showed an increased brain uptake after tariquidar pretreatment in mice, as well as in rats, and exhibited a significant difference between the time-activity curves of the baseline and blocking groups. Biodistribution of both tracers demonstrated a decreased uptake after displacement. [^11^C]SNAP-7941 revealed a high metabolic stability in rats, whereas [^18^F]FE@SNAP was rapidly metabolized.

**Conclusions:**

Both radiotracers demonstrate appropriate imaging properties for the MCHR1. However, the pronounced metabolic stability as well as superior selectivity and affinity of [^11^C]SNAP-7941 underlines the decisive superiority over [^18^F]FE@SNAP.

## Introduction

**Fig. 1:**
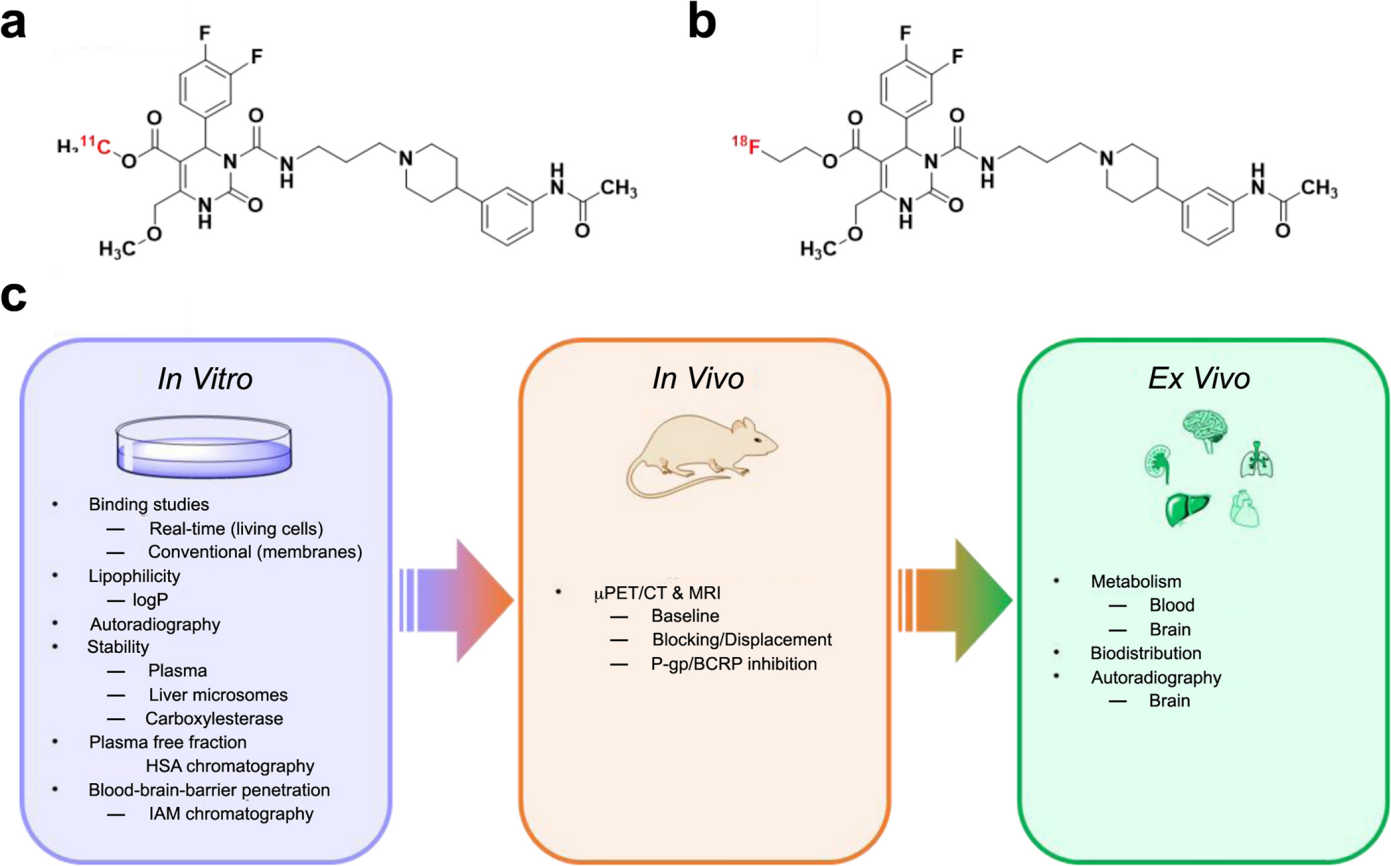
Chemical structures of **a** [^11^C]SNAP-7941 and **b** [^18^F]FE@SNAP and**c** corresponding *in vitro*, *in vivo*, and *ex vivo* experiments. IAM: immobilized artificial membrane; HAS: human serum albumin; P-gp: P-glycoprotein; BCRP: breast cancer resistance protein.

The mammalian melanin-concentrating hormone (MCH), a cyclic 19 amino acid long polypeptide, is primarily produced by neurons in the lateral hypothalamic area (LHA), the incerto-hypothalamic area (IHy), and zona incerta (ZI) [1]. Furthermore, the MCH is also expressed in peripheral organs and tissues, such as the β-cells of the pancreas [2], colonic epithelial cells [3], or adipocytes [4, 5]. The biological function of MCH is mediated by two G-protein coupled receptors (GPCRs): MCH receptor 1 (MCHR1) [6–9] and MCH receptor 2 (MCHR2) [10–13]. The latter has only been found functional in primates, dogs, ferrets, and humans [10, 11]. MCH is playing a key role in the up- and down-regulation of energy homeostasis and body weight. Moreover, MCH/MCHR1 is considered to be involved not only in a variety of pathologies, such as diabetes, insulin resistance, gut inflammation, colitis, and obesity [14–21], but also in a variety of psychiatric disorders, such as depression and anxiety [22]. Furthermore, the MCHR1 is also expressed in the ependymal cells of the ventricular system [23–25], where it acts as an important regulator of cerebrospinal fluid (CSF) flow and cilia beat frequency. As observed in MCHR1 knockout mice, a lack of MCHR1 provokes an increase in ventricular size and could eventually lead to a hydrocephalus [23, 24]. Due to its involvement in a plethora of classical lifestyle diseases, MCHR1 has become a very interesting pharmacological target for clinical medicine, as well as for biomedical research [6–9]. Considering the expression of MCH and MCHR1, the ventricular system, LHA, IHy, ZI, adipose tissue, lung, pancreas, spleen, colon, eyes, as well as muscle tissue are considered primary target regions [2, 3, 6, 11, 23, 24, 26–28]. In the last decade, several MCHR1 antagonists were presented; some have entered clinical trials for the treatment of obesity [29] or are discussed to become anti-diabetic drugs [30]. For quantitative *in vivo* assessment of the MCHR1, a suitable positron emission tomography (PET) tracer is indispensable. A PET tracer would also facilitate preclinical to clinical translation. The specific MCHR1 antagonist SNAP-7941 ((+)-methyl(4S)-3-{[(3-{4-[3-(acetylamino)phenyl]-1piperidinyl}propyl)amino]carbonyl}-4-(3,4-difluorophenyl)-6-(methoxymethyl)-2-oxo-1,2,3,4-tetra-hydro-5-pyrimidenecarboxylate hydrochloride) [31] served as model for the first MCHR1 PET tracers: [^11^C]SNAP-7941 (Fig. 1a), which is the radiolabeled analog of SNAP-7941 and [^18^F]FE@SNAP, a [^18^F]fluoroethylated derivative (Fig. 1b) [32–38].

The current paper contrasts novel and previously attained *in vitro*, *in vivo*, and *ex vivo* assessments of [^11^C]SNAP-7941 and [^18^F]FE@SNAP to determine the superior radioligand with respect to its biological and physicochemical properties. A comprehensive illustration of the applied *in vitro*, *in vivo*, and *ex vivo* experiments is shown in Fig. 1c.

## Materials and Methods

### Chemical Compounds

The racemic mixture of SNAP-7941 ((±)-SNAP-7941), the fluoroethylated analog (±)-(2-fluoroethyl)-3-{[(3-{4-[3-(acetylamino)phenyl]-1-piperidinyl}propyl)amin]carbonyl}-4-(3,4-difluorophenyl)-6-(methoxymethyl)-2-oxo-1,2,3,4-tetra-hydro-5-pyrimidenecarboxylate (FE@SNAP), as well as the precursor compounds (±)-3-{[(3-{4-[3-(acetylamino)phenyl]-1piperidinyl}propyl)amino]carbonyl}-4-(3,4-difluorophenyl)-6-(methoxymethyl)-2-oxo-1,2,3,4-tetra-hydro-5-pyrimidenecarboxylate acid (SNAP-acid), and (±)-2-(tosyloxy)ethyl-3-{[(3-{4-[3-(acetylamino)phenyl]-1piperidinyl}propyl)amino]carbonyl}-4-(3,4-difluorophenyl)-6-(methoxymethyl)-2-oxo-1,2,3,4-tetra-hydro-5-pyrimidenecarboxylate acid (Tos@SNAP) were provided by the Department of Pharmaceutical Chemistry and the Department Organic Chemistry of the University of Vienna (Vienna, Austria) [33]. Tariquidar methanesulfonate (TQD) was purchased from MedChem Express (Princeton, NJ, USA). All other chemicals were purchased from commercial sources and used without further purification.

### Tracer Preparation

The radiosynthesis of [^11^C]SNAP-7941, the radiolabeled analog of (±)-SNAP-7941, was performed in a fully automated synthesizer (TRACERlab™ FX C Pro, GE Healthcare, Germany) as previously reported [30, 33]. Radiosynthesis of [^18^F]FE@SNAP was performed in a microfluidic device (Advion NanoTek®, Ithaca, NY, USA) as described elsewhere [37], followed by purification in a conventional synthesizer unit (Nuclear Interface®, GE Medical Systems, Uppsala, Sweden) [36, 38]. Radiochemical purity and molar activity of [^11^C]SNAP-7941 and [^18^F]FE@SNAP were determined by analytical high-performance liquid chromatography (HPLC) (Agilent Technologies, Santa Clara, CA, USA).

### Kinetic Real-Time Cell-Binding Studies

Kinetic real-time cell-binding studies were performed on adherent Chinese hamster ovary (CHO-K1) cells (negative control) and CHO-K1 cells stably expressing the human MCHR1 or MCHR2 (PerkinElmer®; Waltham, MA, USA) with [^11^C]SNAP-7941 and [^18^F]FE@SNAP as previously reported [37]. The cells were cultured in Ham’s F-12 medium (Gibco®, Life Technologies) with additives (1 % penicillin-streptomycin-glutamine (PSG), 10 % fetal bovine serum (FBS), and 300-μg/m Geneticin (G-418)) and incubated in a humidified 5 % CO_2_ atmosphere at 37 °C. For the preparation of the binding experiment, approximately 10^6^ cells were seeded as a monolayer on the bottom of a tilted cell culture dish (100 mm × 20 mm, CELLSTAR®, Greiner Bio-One) and incubated with 2-ml medium for 24 h to avoid a spreading of the cells over the whole Petri dish. In the next step, the medium was discarded and the Petri dish was placed in a horizontal position with 10-ml medium for additional 24 h. Afterwards, the binding experiment was performed at ambient temperature with LigandTracer® Yellow (Ridgeview Instruments AB, Uppsala, Sweden) using 3-ml fresh medium (Ham’s F-12, serum-free). Unspecific radiotracer uptake was determined with native CHO-K1 cells. The experiments were initiated with baseline measurements for 10–15 min followed by radioligand incubation of [^11^C]SNAP-7941 and [^18^F]FE@SNAP. Binding to the seeded cells was ensured by adding different concentrations of the radiotracer (0.05–1000 nM). Association-time curves were monitored in real-time until the binding equilibrium was achieved. The observed rate constant of the association reaction (*k*_obs_) was determined using non-linear regression curve fitting algorithms implemented in GraphPad Prism 6.0 (GraphPad Software, Inc., San Diego, CA, USA), as previously reported [39, 40]. Cell survival was continuously examined using the perimeter trace (signal *vs.* dish position) of the LigandTracer® 1.0.1 software (Ridgeview Instruments AB, Uppsala, Sweden).

### Plasma Protein Binding Using Bioaffinity Chromatography

The binding of (±)-SNAP-7941 and FE@SNAP to human serum albumin (HSA) was examined by bioaffinity chromatography according to a previously published manuscript [40]. In short, the analytes were diluted in 2-propanol and ammonium acetate buffer (0.5 mg/ml) and injected on the CHIRALPAK®HSA stationary phase (50 × 3 mm, 5 μm pore size, column-batch: H13L-2405, Daicel Chemical Industries, West Chester, PA, USA). Prior the experiments, the column was calibrated with reference standards, and the resulting regression equation was used to convert the logarithmic capacity factors (log(*k′*)) to the percent of plasma protein binding (%PPB). The calibration curves, as well as the experiments, were performed by triplicate injections and at least three times.

### Animals

Ten-week-old male rats (412 ± 58 g, Sprague-Dawley, HIM:OFA, *n* = 30) and 12-week-old male mice (24 ± 6 g, BALB/cAnNRj, *n* = 7) were purchased from the Division of Laboratory Animal Science and Genetics, Himberg, Austria. Animals were kept under conventional housing conditions (22 ± 1 °C; 40–70 % humidity) with food and water supply *ad libitum* and 12-h day/night cycle. All animals were treated according to the European Union rules on animal care and respective animal experiments were approved by the Austrian Ministry of Sciences, Research and Economy (BMWFW-66.009/0029-WF/V/3b/20159). For *in vivo* imaging, animals were anesthetized using 1.5–2 % isoflurane mixed with oxygen (1.5–2 l/min) to avoid movement during the examination. Anesthesia as well as vital parameters were monitored during the time interval of PET acquisition. Radioligands and non-labeled compounds were administered intravenously *via* the lateral tail vein.

### Small Animal Imaging

Anesthetized rats and mice were immobilized in a multimodal animal carrier unit (MACU; medres®—medical research GmbH, Cologne, Germany). The body temperature was preserved at 37 °C throughout the whole experiment. Rats received either [^11^C]SNAP-7941 or [^18^F]FE@SNAP, followed by an injection of (±)-SNAP-7941 (15-mg/kg body weight; displacement study; *n* = 4 for each radiotracer) or the respective solvent serving as the vehicle (baseline condition; *n* = 4 for each radiotracer). The MCHR1 antagonist, (±)-SNAP-7941, and the vehicle were administered either 15 ([^11^C]SNAP-7941) or 20 min ([^18^F]FE@SNAP) after the radiotracer application *via* the lateral tail vein. To investigate a potential binding to the P-glycoprotein (P-gp) and/or breast cancer resistance protein (BCRP) brain efflux transporter system, rats as well as mice were pretreated either with the P-gp/BCRP inhibitor TQD (15 mg/kg body weight; P-gp/BCRP inhibition group; rats: *n* = 4 for each radiotracer; mice: *n* = 4 for [^18^F]FE@SNAP) or the respective solvent (baseline condition; rats: *n* = 3 for each radiotracer; mice: *n* = 3 for [^18^F]FE@SNAP)). Mice received the P-gp/BCRP inhibitor 30 min and rats 60 min before the radiotracer application. Experiments were initiated with a 7-min cone beam attenuation CT (CBCT) of the brain (full rotation, 360 projections; binning 4 × 4; 80 kV; 500 μA; 200-ms exposure time) using a small animal cone beam computed tomography (CBCT) scanner (Siemens Inveon microSPECT/CT, Siemens Medical Solutions, Knoxville, USA). Subsequently, the animals were positioned in the Siemens Inveon microPET scanner (Siemens Medical Solution, Knoxville, USA). The radiotracers were injected intravenously, and dynamic PET imaging was performed for 45 min (rats) in case of [^11^C]SNAP-7941 (77.46 ± 5.41 MBq; molar activity 76.66 ± 23.58 GBq/μmol; radiochemical purity > 99 %) and 30 min (mice)—60 min (rats) for [^18^F]FE@SNAP (47.08 ± 6.24 MBq; molar activity 22.18 ± 9.72 GBq/μmol; radiochemical purity > 90 %).

### Image Reconstruction and Data Post-Processing

Image reconstruction of the CT raw data was performed with a Feldkamp algorithm using a ramp filter followed by standard rat beam-hardening correction and noise reduction (matrix size 1024 × 1024; effective pixel size: 97.56 μm). All CT image data was calibrated to Hounsfield units (HU). PET list mode data were sorted into three-dimensional sinograms according to the following frame sequences, [^11^C]SNAP-7941: 1 × 3 s, 3 × 2 s, 1 × 6 s, 1 × 15 s, 1 × 35 s, 1 × 145 s, 1 × 270 s, 1 × 285 s, 1 × 165 s, 3 × 30 s, 1 × 120 s, 1 × 240 s, 1 × 420 s, 1 × 900 s and for [^18^F]FE@SNAP: 1 × 2 s, 1 × 3 s, 1 × 5 s, 3 × 10 s, 1 × 20 s, 1 × 60 s, 1 × 120 s, 4 × 240 s, 4 × 600 s. PET image reconstruction were performed using an OSEM 3D/OP-MAP with scatter correction and a ramp filter (matrix size 128 × 128). Image data were normalized and corrected for random events, dead time, and radioactive decay. A calibration factor was applied to convert the activity information into absolute concentration units. Multimodal image registration and data post-processing was performed using the biomedical image analysis software PMOD 3.8 (PMOD Technologies Ltd., Zurich, Switzerland) and the Inveon Research Workplace (IRW; Siemens Medical Solutions, Knoxville, USA). Volumes of interest (VOIs) were outlined on the CT images and transferred to the PET data set. Time-activity curves (TACs) were calculated, normalized to dose and weight and expressed as standardized uptake values (SUV; g/ml).

### *Ex Vivo* Biodistribution

After the imaging studies, the animals were sacrificed by decapitation; blood and tissues were removed and collected in tubes, weighed, and subjected to radioactivity measurements in a Gamma Counter (2480 WIZARD^2^, PerkinElmer, Waltham, MA, USA). Values were normalized to the applied dose, the organ, and body weight and expressed as the standardized uptake value (SUV [g/ml]).

### *Ex Vivo* Metabolites

For the analysis of potential radiometabolites, blood samples (50–80 μl) from rats were withdrawn at 10 and 45 min for [^11^C]SNAP-7941 and at 10, 30, and 60 min for [^18^F]FE@SNAP and immediately subjected to the equivalent amount of acetonitrile, homogenized, and stored on ice before processing. Additionally, [^18^F]FE@SNAP was administered to three rats, which were sacrificed after 45 min. Whole brains were harvested and homogenized with equivalent amounts (500–800 μl) of acetonitrile and 0.9 % saline solution using an ULTRA-TURRAX® (T25 basic, IKA Laboratory Equipment, Staufen, Germany). Subsequently, blood and brain were centrifuged (23,000 ×*g*, 4 min, 4 °C; Hettich Universal 30RF, Tuttlingen, Germany), and the obtained supernatant was analyzed by an analytical HPLC equipped with a radioactivity detector (radio-HPLC) (stationary phase: Chromolith® Performance RP-18e, 100–4.6 mm; precolumn: Chromolith® Guard Cartridge RP-18e, 5–4.6 mm; Merck, Darmstadt, Germany), mobile phase: (water/acetic acid 97.5/2.5 *v*/*v*; 2.5 g/l ammonium acetate; pH 3.5)/acetonitrile 70/30 *v*/*v*; flow: 1 ml/min, *λ* = 254 nm). The ratio between metabolite and intact radiotracer was calculated using quantitative HPLC analysis.

### Statistical Analysis

Experimental data are expressed as mean ± SEM of independent experiments (*n* ≥ 3) with different lots of radiolabeled and non-labeled compounds. Statistical testing was performed using GraphPad Prism 7.0 (GraphPad Software, Inc., San Diego, CA). Differences among groups and conditions were determined using either a two-tailed, unpaired Student’s *t* test with Welch’s correction or a two-tailed parametric paired *t* test. Post hoc testing for multiple comparisons was performed using either ordinary one-way ANOVA with Tukey’s correction or ordinary two-way ANOVA with Sidak’s correction. Values of *P* < 0.05 were considered as statistically significant.

## Results

### Kinetic Real-Time Cell-Binding Studies

**Fig. 2:**
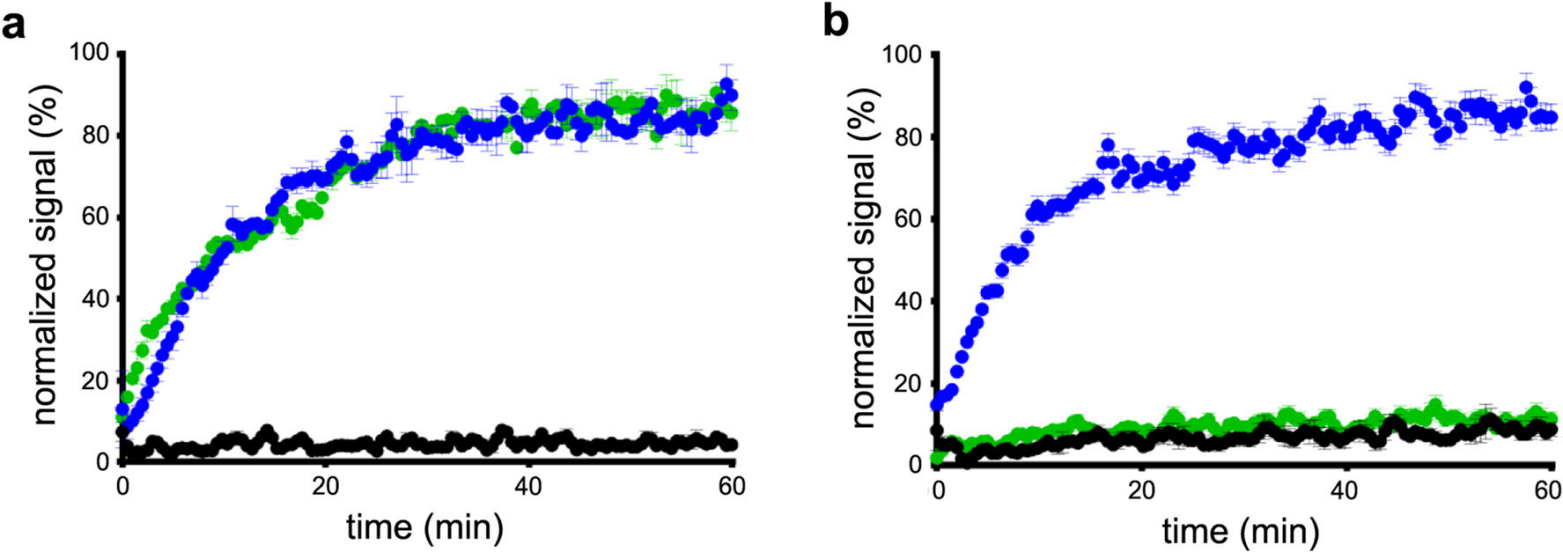
Depiction of the association kinetics of [^11^C]SNAP-7941 (green) and [^18^F]FE@SNAP (blue) on adherent **a** CHO-K1-hMCHR1 and **b** CHO-K1-hMCHR2 cells. The black curve indicates the accumulation of both tracers to native CHO-K1 cells. Data are displayed as mean ± SEM from independent experiments (*n* ≥ 3, each performed as a quadruplicate, 0.05–1000 nM).

Kinetic real-time cell-binding studies were performed in a reliable manner with high temporal resolution as shown in Fig. 2. [^18^F]FE@SNAP and [^11^C]SNAP-7941 demonstrate high accumulation on CHO-K1-hMCHR1 cells, whereas negligible accumulation was detected for the native CHO-K1 cells. The observed association time courses between [^18^F]FE@SNAP (*k*_obs_ = 0.0859 ± 0.0028 min^−1^) and [^11^C]SNAP-7941 (*k*_obs_ = 0.0629 ± 0.0020 min^−1^) on CHO-K1-hMCHR1 cells showed no significant differences (*P* = 0.9). High statistical significance was observed for the accumulation of both radiotracers on the native CHO-K1 cells compared to the CHO-K1-hMCHR1 cells (*P* < 0.0001, Fig. 2a). In contrast, a significant difference in the binding kinetics was observed for the CHO-K1-hMCHR2 cells. The observed association rate constants were significantly different for [^18^F]FE@SNAP (*k*_obs_ = 0.0918 ± 0.0031 min^−1^) compared to [^11^C]SNAP-7941. No significant difference in the binding pattern was determined for [^11^C]SNAP-7941 on the native CHO-K1 cells compared to the CHO-K1-hMCHR2 cells, which indicates negligible accumulation to these two cell lines (Fig. 2b).

### Plasma Protein Binding Using Bioaffinity Chromatography

The PPB analysis of (±)-SNAP-7941 resulted in 80 ± 0.9 % (*n* > 3) and 77 ± 0.2 % (*n* = 3) for FE@SNAP.

### Small Animal Imaging

**Fig. 3:**
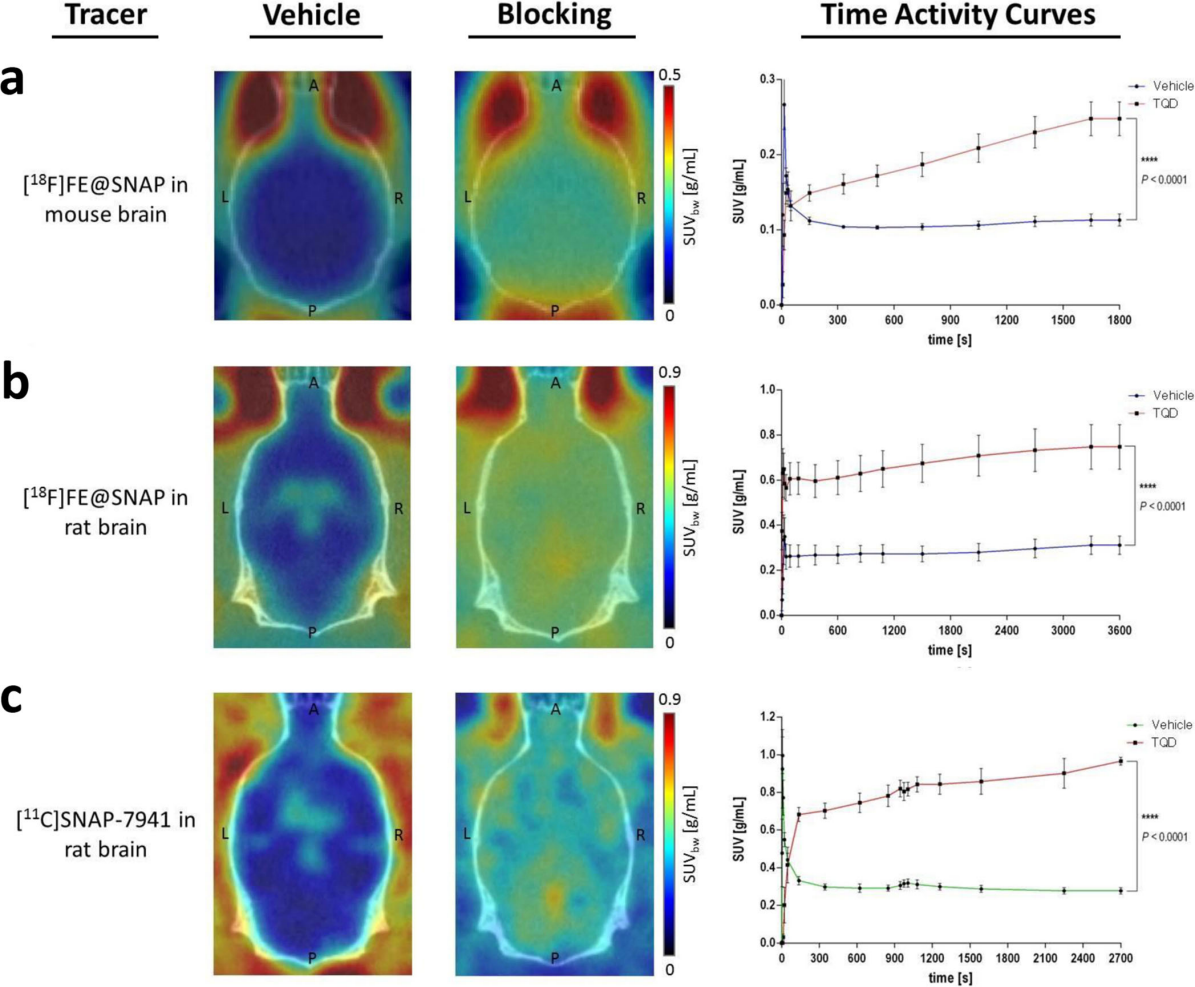
Representation of axial planes of mouse (**a**) and rat (**b**, **c**) brains under vehicle and blocking conditions with the P-gp/BCRP inhibitor TQD for [^18^F]FE@SNAP (**a**, **b**) and [^11^C]SNAP-7941 (**c**). Respective TACs are presented on the right-hand side of each illustration for the dedicated radiotracers [^18^F]FE@SNAP (blue), [^11^C]SNAP-7941 (green) under vehicle conditions, and in combination with 15-mg/kg TQD (red). Data are displayed as mean ± SEM from independent experiments (*n* ≥ 3). Differences among groups were tested using a repeated measures ANOVA (**** = *P* < 0.0001). If not visible, error bars are within the margin of the symbols.

[^18^F]FE@SNAP imaging experiments in mice showed a 1.93-fold increased brain uptake in the TQD blocking group. As illustrated in Fig. 3a, the differences between the TACs of the vehicle and blocking groups were statistically significant (*P* < 0.0001). Furthermore, both radiotracers showed an increased brain uptake for the TQD blocking group in rats. Brain uptake was increased 2.45-times for [^18^F]FE@SNAP (Fig. 3b) and 3.04-times for [^11^C]SNAP-7941 (Fig. 3c), respectively. Both radiotracers exhibited a significant difference between the TACs of the vehicle and blocking groups (*P* < 0.0001).

**Fig. 4:**
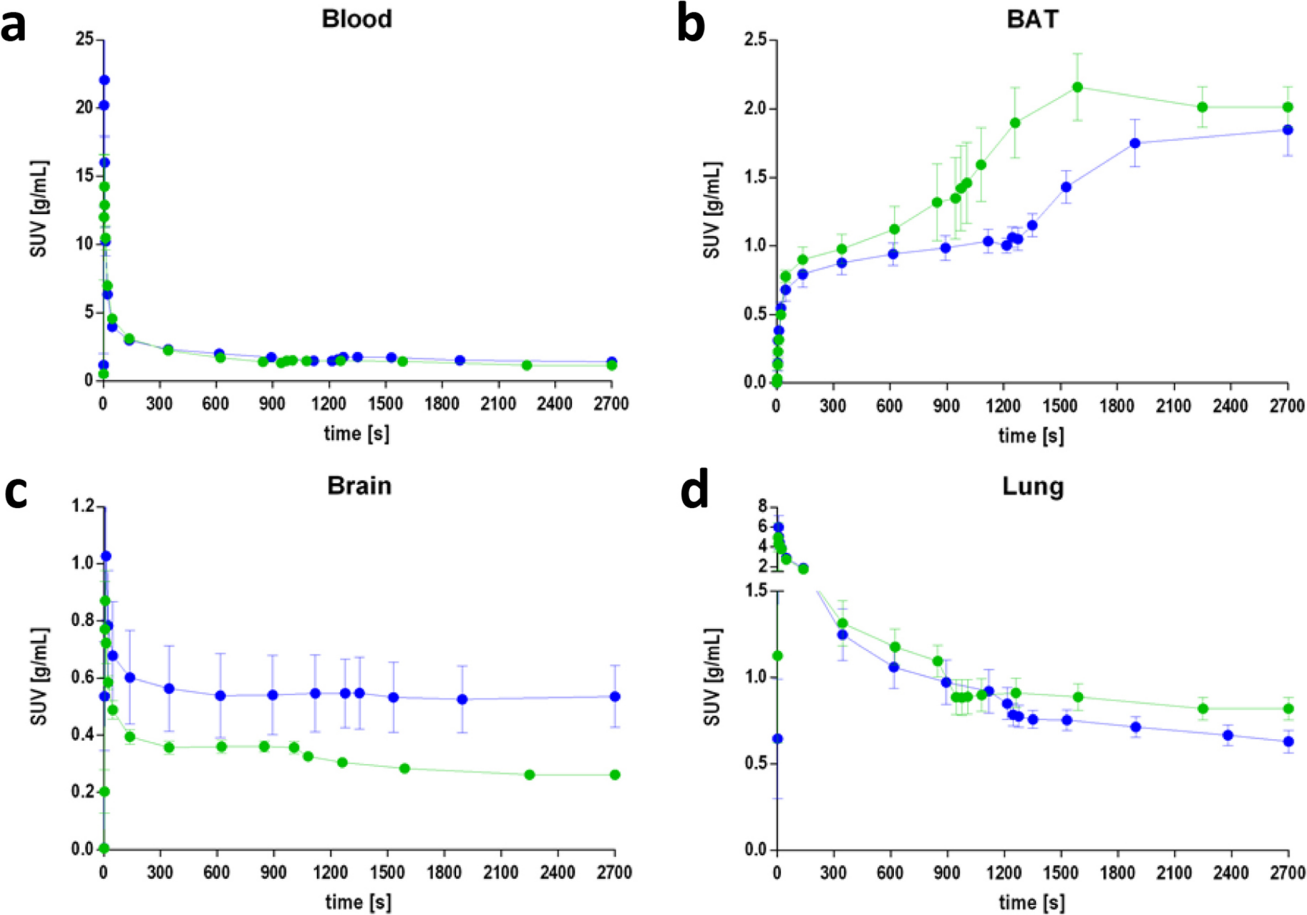
TACs for [^18^F]FE@SNAP (blue) and [^11^C]SNAP-7941 (green) before and after displacement with 15 mg/kg (±)-SNAP-7941 in **a** blood, **b** BAT, **c** brain, and **d** lung, after 900 or 1200 s, respectively. Data are displayed as mean ± SEM from independent experiments (*n* ≥ 3). If not visible, error bars are within the margin of the symbols.

TACs of selected regions were analyzed before and after displacement with 15 mg/kg (±)-SNAP-7941. A clear difference in the binding pattern was observed for both tracers, [^18^F]FE@SNAP and [^11^C]SNAP-7941, for the brown adipose tissue (BAT), brain, and lung. Corresponding TACs are depicted in Fig. 4b–d. The associated blood input curve for both radiotracers is presented in Fig. 4a, showing no significant difference in the uptake behavior (*P* = 0.3117). Differences in the binding profiles before and after displacement with 15 mg/kg (±)-SNAP-7941 in all other regions of interest were not statistically significant, for both [^18^F]FE@SNAP and [^11^C]SNAP-7941.

### *Ex Vivo* Biodistribution

**Fig. 5:**
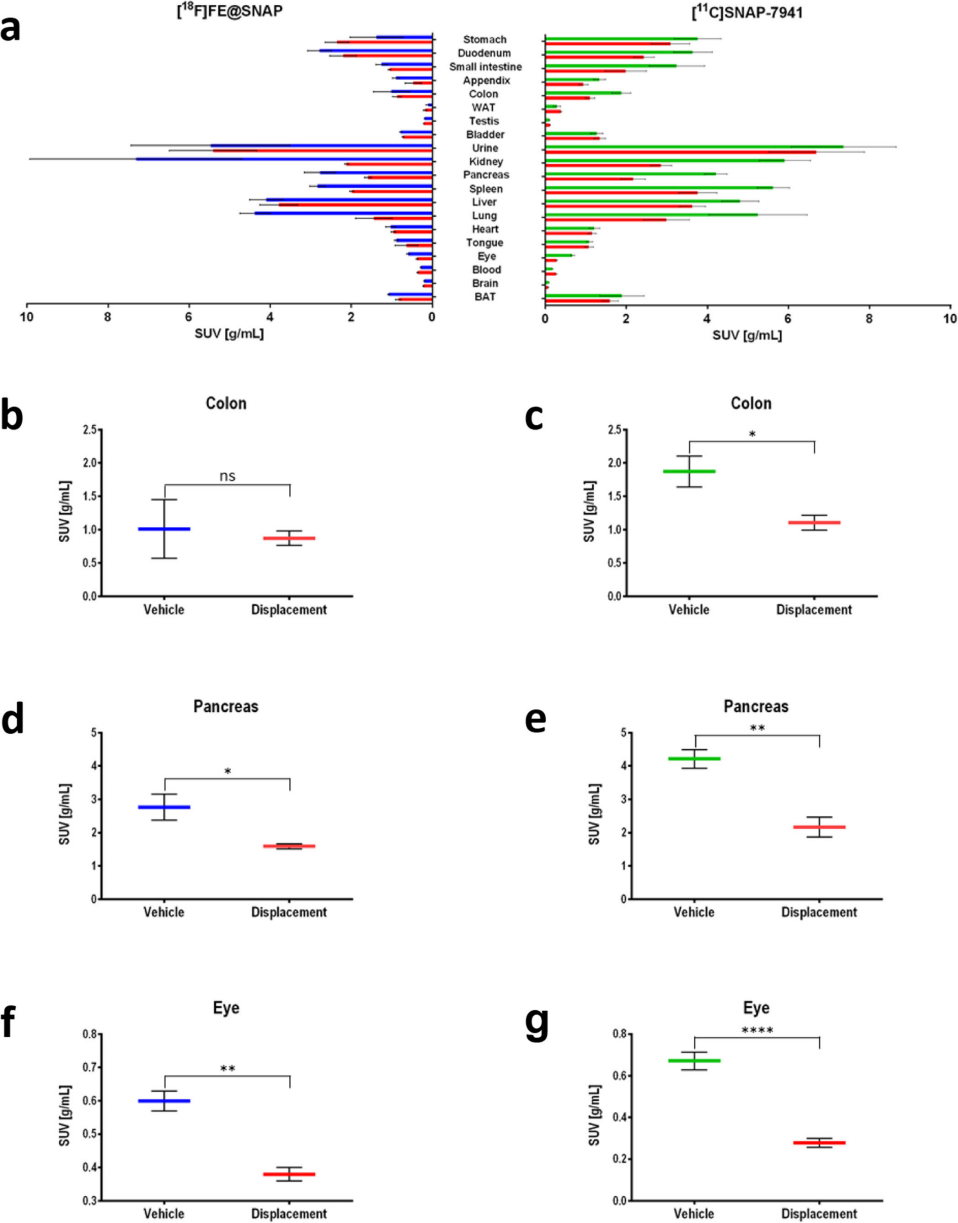
**a** Representative *ex vivo* rat biodistribution of [^11^C]SNAP-7941 and [^18^F]FE@SNAP and **b**–**g** selected target regions of both radiotracers [^18^F]FE@SNAP (blue) and [^11^C]SNAP-7941 (green) under vehicle conditions and after displacement with 15 mg/kg (±)-SNAP-7941 (red). Data are displayed as mean ± SEM from independent experiments (*n* ≥ 3). Differences among groups were tested using a two-tailed parametric paired *t* test (ns = *P* > 0.05; * = *P* < 0.05; ** = *P* < 0.01; **** = *P* < 0.0001). If not visible, error bars are within the margin of the symbols.

The overall biodistribution for both radiotracers demonstrated a decreased uptake in the target regions after displacement with 15 mg/kg (±)-SNAP-7941 (Fig. 5a; [^18^F]FE@SNAP (blue); [^11^C]SNAP-7941 (green); displacement (red)). A detailed statistical analysis was performed for selected target regions (Fig. 5b–g). Statistically significant differences between the vehicle and displacement groups were determined for [^11^C]SNAP-7941 for the colon (*P* = 0.0175; Fig. 5c), pancreas (*P* = 0.0010; Fig. 5e), and eye (*P* < 0.0001; Fig. 5g). In contrast, the colon showed no statistically significant difference for [^18^F]FE@SNAP between the vehicle and displacement groups (*P* = 0.7730; Fig. 5b), whereas the pancreas (*P* = 0.0414; Fig. 5d) and eye (*P* = 0.0037; Fig. 5f) showed significant differences.

### *Ex Vivo* Metabolites

**Fig. 6:**
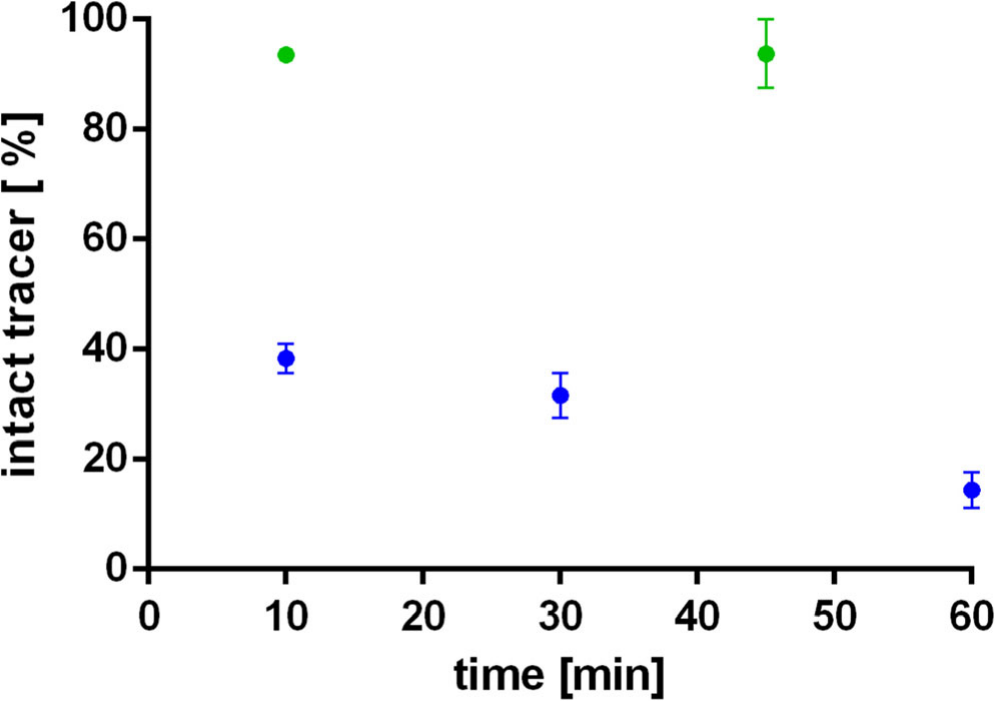
*Ex vivo* metabolic stability of [^18^F]FE@SNAP (blue) and [^11^C]SNAP-7941 (green) in rat whole blood over time. Data are displayed as mean ± SEM from independent experiments (*n* ≥ 3). If not visible, error bars are within the margin of the symbols.

Ten minutes after the radiotracer application, 38.40 ± 2.3 % of intact [^18^F]FE@SNAP was present in rat whole blood; at 30 min after administration, 31.59 ± 4.0 % was left and 14.42 ± 3.3 % at 60 min. Moreover, the formation of a radioactive hydrophilic metabolite was observed. On the contrary, [^11^C]SNAP-7941 evinced a high metabolic stability in rat whole blood, resulting in 93.52 ± 0.1 % of intact tracer at 10 min and 93.74 ± 6.2 % at 45 min (Fig. 6). The investigation of brain metabolites evinced a strong degradation of the parent compound at 45 min (22.37 ± 5.8 % of intact tracer) for [^18^F]FE@SNAP.

## Discussion

To quantify the biomolecular mechanisms of the MCHR1 *in vivo*, a selective PET radioligand is indispensable. Hence, a specific MCHR1 PET-tracer would provide deeper insights on the receptor’s involvement in lifestyle diseases, such as obesity and diabetes, and promote drug development for related pathologies. Nevertheless, only three PET radioligands for the visualization of the MCHR1 have been developed [32, 36, 41, 42]. This paper focuses on the first MCHR1 PET ligands, [^18^F]FE@SNAP and [^11^C]SNAP-7941, and contrasts their advantages and disadvantages. Table 1 gives an overview of the results, which are discussed in this section.

**Table 1.** Overview of discussed results

		[^11^C]SNAP-7941		[^18^F]FE@SNAP	
*In vitro*	Radiosynthesis	2.9 ± 1.6 GBq	[33]	374 ± 202 MBq	[34]
	Affinity to MCHR1 (*K*_I_)	3.91 ± 0.74	[25]	9.98 ± 1.12	[25]
	Selectivity over MCHR2	Yes		No	
	Lipophilicity (log*D*)	3.29 ± 0.01	[30]	3.83 ± 0.1	[34]
	Plasma free fraction (human)	20.96 ± 1 %	[30]	12.6 ± 0.2 %	[34]
	Binding to human serum albumin	80 ± 0.9 %		77 ± 0.2 %	
	Autoradiography	✓	[32]	✓	[32]
*In vivo*	Small animal PET	✓		✓	
*Ex vivo*	Biodistribution	✓		✓	
	Metabolism in rat blood	Stable		Rapidly metabolized	
	Metabolism in rat brain	Stable		Rapidly metabolized	

Regarding the synthesis of these two tracers, [^11^C]SNAP-7941 is the more reliable compound, due to its higher yields (2.9 ± 1.6 GBq *vs.* 374 ± 202 MBq for [^18^F]FE@SNAP) and fewer preparation steps. Moreover, its radiosynthesis is faster (40 min) compared to [^18^F]FE@SNAP (100 min), which has to be synthesized *via* a microfluidic device [35, 36].

Since MCHR2 is not expressed in rodents, additional kinetic real-time cell-binding studies were performed to substantiate the target selectivity. In this context, both radiotracers demonstrated a specific accumulation profile on the CHO-K1-hMCHR1 cells and negligible accumulation on the native CHO-K1 cells (Fig. 2a). While [^11^C]SNAP-7941 evinced selective binding to the CHO-K1-hMCHR1 cells, which is in good agreement with previously published data [32], [^18^F]FE@SNAP additionally exhibited accumulation to the CHO-K1-hMCHR2 cells (Fig. 2b). This phenomenon contradicts previously elaborated findings on CHO-K1-hMCHR2 membranes [34] and might be explained by the difference in the biochemical approach (competition experiments with the unlabeled ligand *vs.* direct binding with the radiolabeled ligand) and the experimental setup (membranes *vs.* living cells). In this context, it has to be highlighted that experiments on living cells, as performed in the present study, enhance the understanding of the complex interplay between the radiotracer and the dedicated biological target [39]. Moreover, previous experiments revealed higher binding affinity for (±)-SNAP-7941 (*K*_i_ = 3.91 ± 0.74 nM) compared to FE@SNAP (*K*_i_ = 9.98 ± 1.12 nM) [25]. Based on current and previous results, [^11^C]SNAP-7941 exhibits an improved target affinity and superior selectivity.

Considering the *in vivo* pharmacology, it has been demonstrated in preceding [32, 43] and recent experiments that [^11^C]SNAP-7941 is a P-gp/BCRP substrate, as confirmed in small animal imaging studies of rat brains (3.04-times higher uptake after TQD pretreatment, Fig. 3c). A similar behavior was observed for [^18^F]FE@SNAP (2.45-times higher uptake after TQD pretreatment, Fig. 3b). Additionally, no species differences in P-gp/BCRP inhibition between mouse and rat were found as shown in small animal imaging studies of mouse brains with [^18^F]FE@SNAP, revealing also an increased brain uptake after TQD pretreatment (Fig. 3a). Detailed quantitative assessment of the whole brain uptake evinced a more distinct difference between the vehicle and TQD-treated groups for [^11^C]SNAP-7941 unlike [^18^F]FE@SNAP. These variations result from the already higher brain uptake in the vehicle group of [^18^F]FE@SNAP, which presumably originates from a higher unspecific binding. This stands in line with the physicochemical parameters already determined in previous studies showing a lower log*D* (3.29 ± 0.01) for [^11^C]SNAP-7941 compared to [^18^F]FE@SNAP (log*D* = 3.83 ± 0.1). In previous studies, plasma free fractions of 12.6 ± 0.2 % were reported for [^18^F]FE@SNAP and of 20.96 ± 1 % for [^11^C]SNAP-7941 using an ultrafiltration method with human pooled plasma [32, 36]. For (±)-SNAP-7941, these results were in accordance with the bioaffinity chromatography outcome, indicating that (±)-SNAP-7941 utterly binds to serum albumin. Whereas, the results for FE@SNAP diverged around 10 % when applying the chromatographic method. One reason might be that FE@SNAP also binds to other plasma components, such as alpha1-acid glycoprotein or diverse lipoproteins.

Furthermore, the higher brain uptake of [^18^F]FE@SNAP compared to [^11^C]SNAP-7941 results from the formation of radiometabolites (*e.g.*, [^18^F]fluoroethanol) supported by the findings from the *ex vivo* metabolism studies in rat whole blood (Fig. 6), which underlines the superior imaging contrast of [^11^C]SNAP-7941 in the brain.

Considering the vehicle and displacement groups, the analysis of the whole brain resulted in a clear displacement for [^11^C]SNAP-7941, whereas for [^18^F]FE@SNAP a displacement could not be detected (Fig. 4c). This finding is supported by previously conducted experiments [25] and is likely a result of the high unspecific binding and metabolic degradation of [^18^F]FE@SNAP. Furthermore, a drop in the TAC of both radiotracers after displacement with 15 mg/kg (±)-SNAP-7941 could be detected for the lung (Fig. 4d). The detailed analysis of the TACs of other target regions, such as tongue, pancreas, and colon, was not possible due to the limited field of view of the imaging modality and spillover effects of the surrounding tissue. Interestingly, the TAC of the BAT depicts an increased uptake for both tracers after the administration of (±)-SNAP-7941 (Fig. 4b), which indicates a potential involvement of the MCHergic system and a further interaction of important regulatory pathways. The representative TAC of the blood pool confirms the proper administration and further bioavailability of both radiotracers (Fig. 4a).

Global analysis of the *ex vivo* biodistribution depicts reduced tracer uptake after displacement with (±)-SNAP-7941 for both radiotracers (Fig. 5a). This finding is highlighted by previously conducted studies [38] and confirmed by tissue-to-blood analyses [32]. The reduced uptake in BAT stands in contrast to the enhanced uptake shown in the TAC (Fig. 4b). This phenomenon may be explained by the differences in the experimental setup due to the high biodiversity when using different animals as used in biodistribution studies. In contrast, the *in vivo* displacement analysis of the TACs was performed within the same animal. The involvement of the MCHR1 in BAT is part of ongoing investigations.

Regional analysis of selected target regions (colon, pancreas, eye) contrasts the higher suitability of [^11^C]SNAP-7941. Even if both radiotracers demonstrate a significant decrease in uptake in the pancreas and eye after displacement with (±)-SNAP-7941, [^11^C]SNAP-7941 revealed a more pronounced difference. However, a significant displacement in the colon was only observed for [^11^C]SNAP-7941 (Fig. 5b–g); a possible explanation might be the fast and high metabolic degradation of [^18^F]FE@SNAP, which hampered the assessment of the as due to the high metabolic degradation of [^18^F]FE@SNAP no significant displacement.

Furthermore, [^11^C]SNAP-7941 evinced a high metabolic stability in rat whole blood, whereas [^18^F]FE@SNAP was rapidly metabolized (Fig. 6), which was validated by previous *in vitro* as well as *in vivo* studies. A detailed analysis of the brain metabolism demonstrated an extensive degradation of [^18^F]FE@SNAP, while [^11^C]SNAP-7941 remained metabolically stable [32, 36, 38].

Even though both tracers exhibit suitable properties for the imaging of the MCHR1, [^11^C]SNAP-7941 clearly demonstrated superior imaging properties due to its higher selectivity, affinity, and metabolic stability. Based on the combined data, we recommend [^11^C]SNAP-7941 as the tracer of choice for the imaging of MCHR1.

## Conclusions

The MCHR1 has become an interesting pharmacological target for clinical medicine, as well as for biomedical research, since it may be involved in a plethora of lifestyle diseases. In this context, the availability of a suitable PET radiotracer is a crucial step for the quantitative *in vivo* assessment of MCHR1 pharmacology. Extensive *in vitro*, *in vivo*, and *ex vivo* assessments of [^18^F]FE@SNAP and [^11^C]SNAP-7941 demonstrate appropriate imaging properties for the MCHR1. Yet, some physiological processes influenced by the MCH system, as for instance its contribution to BAT stimulation, remain unclear and demand further elucidation.

However, the pronounced metabolic stability as well as superior affinity and selectivity of [^11^C]SNAP-7941 reveal the decisive superiority over [^18^F]FE@SNAP. Since humans express both the MCHR1 and MCHR2, tracer selectivity is essential for prospective first-in-man imaging studies. Therefore, [^11^C]SNAP-7941 is the ideal candidate for initial clinical trials, addressing the imaging of endocrinological and psychiatric disorders.
